# Retraction Note To: Functional activation of Proline-rich Tyrosine Kinase2 (PYK2) in peripheral blood mononuclear cells from patients with systemic lupus erythematosus

**DOI:** 10.1186/s12891-015-0462-0

**Published:** 2015-06-19

**Authors:** Meiying Wang, Hongsheng Sun, Wei Zhang, Yuanchao Zhang

**Affiliations:** Department of Rheumatology and Immunology, Provincial Hospital affiliated to Shandong University, Jinan, 250021 China; Department of Pain Management, Hospital affiliated to Medical College of Qingdao University, Qingdao, 266003 China

## Retraction

This article [1] has been retracted by the editors of BMC Musculoskeletal Disorders due to extensive overlap with previously published work [2]. We apologise for the inconvenience caused.
